# A Rare, Atypical Presentation of Decompensated Cirrhosis: Isolated Transudative Hepatic Chylothorax Without Ascites

**DOI:** 10.1155/crhe/5596329

**Published:** 2025-10-06

**Authors:** Michael Yulong Wu, Rhian Aghajani, Sophie Timmins, Anna Di Bartolomeo, Rachael Jacob, Robert Ng, Jacob George, Harry Crane, Cameron Gofton

**Affiliations:** ^1^Department of Gastroenterology and Hepatology, Royal North Shore Hospital, Sydney, New South Wales, Australia; ^2^Northern Clinical School, University of Sydney, Sydney, New South Wales, Australia; ^3^Department of Respiratory Medicine, Royal North Shore Hospital, Sydney, New South Wales, Australia; ^4^Department of Gastroenterology and Hepatology, Westmead Hospital, Sydney, New South Wales, Australia; ^5^Westmead Institute of Medical Research, Sydney, New South Wales, Australia; ^6^A. W. Morrow Gastroenterology and Liver Centre, Royal Prince Alfred Hospital, Sydney, New South Wales, Australia; ^7^Department of Radiology, Royal North Shore Hospital, Sydney, New South Wales, Australia

## Abstract

Hepatic hydrothorax is an uncommon presentation of decompensated liver cirrhosis and usually presents after other complications of portal hypertension such as ascites. We report the case of a 73-year-old female with autoimmune hepatitis (AIH) treated with budesonide, presenting with a right-sided hepatic chylothorax secondary to AIH and subsequent diagnosis of primary biliary cholangitis (PBC). Pleural fluid analysis revealed a transudative chylothorax, whilst serology, liver elastography, hepatic venous pressure gradients and biopsy diagnosed advanced fibrosis with portal hypertension secondary to AIH–PBC overlap syndrome. Commencement of diuretics led to the resolution of the recurrent pleural effusion. Chylothorax is typically an exudative effusion; however, in very rare cases of decompensated liver cirrhosis, it may present as an isolated transudative effusion in the absence of other signs of portal hypertensive complications such as ascites. This is the first reported case of decompensated cirrhosis secondary to AIH–PBC overlap syndrome, presenting as an isolated unilateral transudative chylothorax.

## 1. Introduction

Hepatic hydrothorax is an uncommon manifestation of decompensated liver cirrhosis [1]. It is typically due to portal hypertension from advanced liver cirrhosis; diagnosis is made in the absence of alternative cardiopulmonary, renal or malignant pathology. Hydrothorax often occurs in the context of significant ascites and, in rare cases, has been described as the initial sign of decompensated liver cirrhosis.

We present the case of a patient presenting with recurrent isolated right-sided transudative chylothorax on a background of autoimmune hepatitis (AIH) managed with oral budesonide. After extensive investigations, a diagnosis of hepatic chylothorax secondary to AIH and primary biliary cholangitis (PBC) overlap syndrome was established. This case underscores the importance of considering hepatic hydrothorax as a potential cause of transudative pleural effusions, even in the absence of other complications of portal hypertension such as ascites. Additionally, we highlight the need to switch to steroid-sparing agents upon any new diagnosis of portal hypertension to avoid the systemic side effects associated with budesonide use.

## 2. Case Report

A 73-year-old female presents to the hospital with 2 weeks of progressive exertional dyspnoea. Three months prior, she had presented with a right-sided transudative pleural effusion requiring catheter drainage and had remained well on discharge. During this preceding admission, the patient was switched from prednisolone and azathioprine to oral budesonide for management of AIH due to recent osteoporotic fractures and leukopaenia. Medical history included prior heart failure with preserved ejection fraction, severe osteoporosis with vertebral fractures and AIH.

On examination, the patient was tachypnoeic with oxygen desaturations. Jugular venous distention was mildly elevated. On auscultation, there was reduced air entry with stony dull percussion of the right lung. No murmurs were heard on auscultation. The abdomen was soft and nontender. There was minor pitting oedema in the peripheries. There were no peripheral stigmata of chronic liver disease.

Laboratory investigations showed haemoglobin 99 g/L, normal white cell count, platelet count and no renal impairment. There were new mixed liver test derangements with bilirubin 58 µmol/L, alkaline phosphatase 196 u/L, gamma-glutamyl transferase 370 u/L, alanine aminotransferase 237 u/L and aspartate aminotransferase 165 u/L. N-terminal pro-brain natriuretic peptide (BNP) was 388 ng/L.

**Figure 1:**
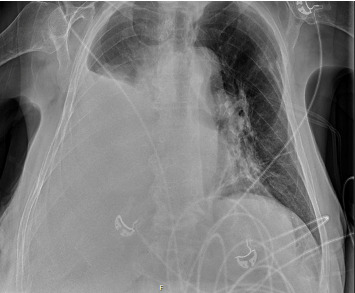
Chest X-ray showed a right-sided pleural effusion tracking to the apex with associated complete lower and middle lobe atelectasis, as well as partial atelectasis of the right upper lobe.

**Figure 2:**
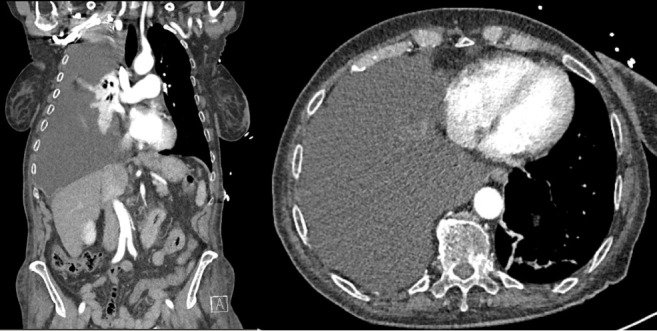
CT chest with unilateral pleural effusion.

A chest X-ray and CT of the chest, abdomen and pelvis demonstrated a large unilateral right-sided pleural effusion with mediastinal mass effect and a homogenous liver without evidence of nodularity or splenomegaly (Figures 1 and 2). A chest drain was inserted with an initial drainage of 2500 mL of opaque milky fluid with persistent high output. Pleural fluid analysis was consistent with a transudative effusion by Light's criteria (serum: pleural protein 69 g/L:15 g/L, serum: pleural lactate dehydrogenase 590:90 u/L, upper limit serum lactate dehydrogenase 250 u/L). No malignant cells were seen on cytology. Further analysis demonstrated a high pleural fluid triglyceride level of 3.2 mmol/L (283.2 mg/dL), low cholesterol of 0.9 mmol/L (34.8 mg/dL) and the presence of chylomicrons consistent with chylothorax. Transthoracic echocardiogram showed normal systolic function with an ejection fraction of 60%, normal valvular flow and normal right ventricular and diastolic function. At the time, differential diagnoses considered were mediastinal malignancy with thoracic duct disruption or decompensated heart failure. However, given the normal echocardiogram, low serum BNP, unilateral chylothorax and only mild peripheral examination findings, the cardiology team felt that exacerbation of heart failure was inconsistent with the aetiology of the effusion. Hepatic hydrothorax was initially considered; however, they also felt unlikely in the context of early fibrosis on previous liver biopsy, lack of imaging findings consistent with progression to cirrhosis and no portal hypertensive complications such as ascites, splenomegaly or thrombocytopaenia.

Further investigation with lymphoscintigraphy with SPECT/CT did not show a thoracic duct leak or diaphragmatic defect. An FDG-PET scan did not identify any FDG-avid malignancy. Subsequent liver elastography revealed a median stiffness of 23.9 kPa and controlled attenuation parameter 185 dB/m consistent with F4 fibrosis/cirrhosis. Transjugular liver biopsy demonstrated a hepatic venous pressure gradient (HVPG) of 12 mmHg. Liver biopsy revealed chronic active hepatitis with bile duct injury and prominent bridging fibrosis consistent with F3 fibrosis. An extended autoimmune liver panel detected antibodies to Sp100 and anti-centromere protein B.

The patient was diagnosed with AIH and PBC overlap syndrome with an initial presentation of isolated hepatic chylothorax. Ursodeoxycholic acid and mycophenolate mofetil were commenced and gradually uptitrated. Budesonide was changed to prednisolone. Regular frusemide and spironolactone diuretic dosages were increased. There were significant clinical improvements with the resolution of the recurrent pleural effusion. Long-term follow-up showed resolution of liver function tests and no recurrence of hepatic chylothorax.

## 3. Discussion

Isolated hepatic hydrothorax in the absence of ascites is rare and can pose significant diagnostic challenges. Hepatic hydrothorax only occurs in 5%–15% of patients with cirrhosis, but fewer than 10% of presentations occur in the absence of ascites or other sequelae of portal hypertension [2]. The condition becomes even more uncommon when the pleural effusion is transudative and chylous in nature. As in our case, chylothorax is diagnosed with the identification of chylomicrons in the pleural fluid by electrophoresis, or when pleural fluid triglyceride levels exceed 110 mg/dL, cholesterol levels are below 200 mg/dL, and the ratio of pleural to serum triglycerides is greater than 1. Chylothorax is typically an exudative effusion; however, a transudative chylothorax can occur in the setting of nephrotic syndrome, heart failure and, in 1% of cases, liver cirrhosis [3]. There are only seven previously described cases of transudative chylothorax in the setting of liver cirrhosis due to hepatitis C, alcohol misuse and two cryptogenic cases [3–9]. We report the first case of an AIH and PBC overlap presenting as isolated transudative chylothorax in the setting of advanced liver fibrosis.

Diagnosis of hepatic chylothorax may be overlooked in the absence of a prior diagnosis of cirrhosis or ascites and is established by exclusion of alternative causes of pleural effusion. Initial investigations should include imaging with echocardiogram and CT chest, pleural fluid analysis with cytology and thoracoscopy or pleural biopsy if there is suspicion for malignancy. One proposed mechanism for the pathogenesis of hepatic hydrothorax involves rupture of pleuroperitoneal blebs under increased intra-abdominal pressure, resulting in diaphragmatic defects [10]. During inspiration, negative intrathoracic pressure facilitates unidirectional movement of ascitic fluid from the peritoneal cavity into the pleural space, functioning like a one-way valve and in some cases, leading to the absence of detectable ascites. When results are equivocal, more advanced techniques with nuclear medicine scintigraphy and MRI may be considered [1, 10, 11]. Lymphoscintigraphy with SPECT/CT in this case did not indicate tracer migration to localise a responsible diaphragmatic defect or a thoracic duct leak. We highlight the importance of maintaining high clinical suspicion for portal hypertension with a low threshold for transjugular liver biopsy and HVPG measurements when investigations do not yield a diagnosis. In this case, the HVPG was elevated; normal values are typically ≤ 5 mmHg, while portal hypertension is defined as > 5 mmHg and clinically significant portal hypertension as ≥ 10 mmHg. In the absence of other causes, the hepatic chylothorax was most likely driven by portal hypertension. Initial treatment of this would include a combination of sodium restriction, low-fat high-protein diet, diuretics and thoracocentesis as needed. In refractory cases, consideration for transjugular intrahepatic portosystemic shunt placement or referral for liver transplantation may be considered. However, these were relatively contraindicated given the prior history of heart failure and patient's advanced age.

In this case, budesonide exposure in the setting of unrecognised portal hypertension may have contributed to the history of osteoporotic fractures and places the patient at higher risk of infections. Budesonide has a hepatic first-pass effect of 90%, which is impaired in portal hypertension, resulting in elevated systemic cortisol levels and suppression of the hypothalamic–pituitary–adrenal axis [12–14]. An earlier diagnosis of portal hypertension could have reduced systemic steroid exposure and prevent possible risk of complications such as hepatic vein thromboembolism, infections and osteoporosis [12]. This highlights the importance of promptly diagnosing portal hypertension to enable safe medication management and reduce associated risks.

## 4. Conclusion

We report the first case of an AIH and PBC overlap syndrome presenting as isolated hepatic chylothorax. Clinicians should be reminded that hepatic chylothorax can present as transudative effusions, in the absence of ascites, and therefore, underlying portal hypertension should always be considered in the differential diagnosis. Once underlying cirrhosis is diagnosed in patients with AIH, budesonide should be avoided due to the risks of systemic side effects from portosystemic shunts.

## Data Availability

The data that support the findings of this study are available from the corresponding author upon reasonable request.

## References

[1] Garbuzenko D. V., Arefyev N. O. (2017). Hepatic Hydrothorax: An Update and Review of the Literature. *World Journal of Hepatology*.

[2] Badillo R., Rockey D. C. (2014). Hepatic Hydrothorax: Clinical Features, Management, and Outcomes in 77 Patients and Review of the Literature. *Medicine*.

[3] Owda F., Mallah S., Ayyad M. (2023). A Very Uncommon Case of Transudative Chylothorax: A Case Report and Literature Review. *Cureus*.

[4] Mirza T. M., Ali R., Golubykh K., Malik M., Kovalenko I., Lippold C. Transudative Chylous Pleural Effusion and Chylous Ascites: A Rare Manifestation of Liver Cirrhosis.

[5] Nganga R., Pulliam C., Sessions W., Stola A., Gregg J. (2022). Transudative Chylothorax in a Liver Cirrhosis Patient: A Case Report. *Respiratory Medicine Case Reports*.

[6] Gukasyan J., Phan A. T., Hu J. (2023). Left-Sided Transudative Chylothorax With Concomitant Chylous Ascites in the Setting of Liver Cirrhosis. *Cureus*.

[7] Koller A. Z., Bhalani N., Matheus M., Mohan K. (2023). S3804 Hepatic Chylothorax in the Absence of Ascites: A Rare Pleural Effusion. *American Journal of Gastroenterology*.

[8] Akbar A., Hendrickson T., Vangara A., Marlowe S., Hussain A., Ganti S. S. (2023). Hepatic Chylothorax: An Uncommon Pleural Effusion. *Journal of Investigative Medicine High Impact Case Reports*.

[9] Migaou A., Ben Saad A., Baili H. (2020). Transudative Chylothorax in Liver Cirrhosis; An Underappreciated Entity. *Respiratory Medicine Case Reports*.

[10] Roussos A., Philippou N., Mantzaris G. J., Gourgouliannis K. I. (2007). Hepatic Hydrothorax: Pathophysiology Diagnosis and Management. *Journal of Gastroenterology and Hepatology*.

[11] Pippard B., Bhatnagar M., McNeill L., Donnelly M., Frew K., Aujayeb A. (2022). Hepatic Hydrothorax: A Narrative Review. *Pulmonary Therapy*.

[12] Manns M. P., Jaeckel E., Taubert R. (2018). Budesonide in Autoimmune Hepatitis: The Right Drug at the Right Time for the Right Patient. *Clinical Gastroenterology and Hepatology*.

[13] Geier A., Gartung C., Dietrich C. G., Wasmuth H. E., Reinartz P., Matern S. (2003). Side Effects of Budesonide in Liver Cirrhosis Due to Chronic Autoimmune Hepatitis: Influence of Hepatic Metabolism Versus Portosystemic Shunts on a Patient Complicated with HCC. *World Journal of Gastroenterology*.

[14] Hempfling W., Grunhage F., Dilger K., Reichel C., Beuers U., Sauerbruch T. (2003). Pharmacokinetics and Pharmacodynamic Action of Budesonide in Early- and Late-Stage Primary Biliary Cirrhosis. *Hepatology*.

